# Anti-B Cell Maturation Antigen Chimeric Antigen Receptor T Cell Therapy for the Treatment of AL Amyloidosis and Concurrent Relapsed/Refractory Multiple Myeloma: Preliminary Efficacy and Safety

**DOI:** 10.3390/curroncol30110697

**Published:** 2023-10-31

**Authors:** Saurav Das, Sikander Ailawadhi, Taimur Sher, Vivek Roy, Andre Fernandez, Ricardo D. Parrondo

**Affiliations:** 1 Division of Hematology-Oncology and Blood and Marrow Transplantation Program, Mayo Clinic, Jacksonville, FL 32224, USA; das.saurav@mayo.edu (S.D.); ailawadhi.sikander@mayo.edu (S.A.); sher.taimur@mayo.edu (T.S.); roy.vivek@mayo.edu (V.R.); fernandez.andre@mayo.edu (A.F.); 2 Mangurian Building, 3rd Floor, 4500 San Pablo Road S, Jacksonville, FL 32224, USA

**Keywords:** Immunotherapy, multiple myeloma, AL amyloidosis, CAR-T

## Abstract

While immunotherapies, such as CAR T therapy and bi-specific antibodies, have revolutionized the treatment of multiple myeloma (MM), patients with AL amyloidosis have been excluded from trials with these agents due to concerns of underlying autonomic, cardiac, and renal dysfunction, leading to potentially fatal toxicities from these therapies. In this communication, we described the outcomes of two patients with AL amyloidosis and concurrent MM with underlying cardiac and/or renal dysfunction who underwent anti-BCMA CAR T cell therapy with ide-cel or cilta-cel, received cytokine release syndrome prophylaxis, and tolerated therapy well with manageable toxicities and achieved a MRD-negative state. We described the preliminary efficacy and safety of CAR T in patients with AL amyloidosis and highlighted the importance of patient selection and medical optimization of cardiac and renal function prior to CAR T.

## 1. Introduction

AL amyloidosis is a plasma cell dyscrasia, in which monoclonal immunoglobulin light chains secreted by clonal plasma cells deposit in extracellular tissues as insoluble fibrils and cause organ damage [1,2]. The advent of novel therapies, such as proteasome inhibitors (PIs), immunomodulatory drugs (IMiDs), anti-CD38 monoclonal antibodies (MoAbs) and the use of high-dose melphalan followed by autologous stem cell transplantation (ASCT) have led to an improvement in the survival outcomes of patients with AL amyloidosis [3]. Additionally, the front-line use of daratumumab-bortezomib-cyclophosphamide-dexamethasone (Dara-CyBorD) has led to higher and more durable hematologic, cardiac, and renal response rates compared to the previous standard of care, CyBorD [4]. However, AL amyloidosis is incurable and relapsed and refractory disease is common, as is morbidity and mortality related to light chain deposition and subsequent organ dysfunction. B cell maturation antigen (BCMA) is a protein belonging to the tumor necrosis factor superfamily that regulates B cell proliferation, survival, as well as maturation into plasma cells [5]. Targeting BCMA via the use of cellular immunotherapies, such as chimeric antigen receptor (CAR) T cells or bi-specific antibodies has led to unprecedented response rates in patients with relapsed/refractory multiple myeloma [6,7,8]. However, patients with AL amyloidosis were excluded from these trials with BCMA-directed cellular immunotherapies, and there is a serious concern about the physiologic tolerability of some of the toxicities of these therapies, such as cytokine release syndrome (CRS) or immune effector cell-associated neurotoxicity syndrome (ICANS), as patients with amyloid-induced cardiac and renal dysfunction may not have enough organ reserve to safely tolerate these toxicities [9]. There is a rationale to target BCMA in patients with AL amyloidosis, given that BCMA is highly expressed on amyloidogenic plasma cells [10]. Additionally, high response rates have been seen with the BCMA-directed antibody drug conjugate belantamab mafodotin in patients with relapsed, systemic AL amyloidosis [11]. Medical optimization of organ function, as well as CRS prophylaxis, may mitigate CAR T toxicity and may possibly make CAR T cell therapy a safe therapeutic strategy for patients with AL amyloidosis. Herein, we described the outcomes of two patients with primary systemic AL amyloidosis with coexistent multiple myeloma who had medical optimization of cardiac and renal function, received corticosteroid CRS prophylaxis, and subsequently underwent anti-BCMA CAR T cell therapy with manageable toxicity and achievement of a minimal residual disease (MRD)-negative state.

## 2. Case 1

A 62-year-old Caucasian woman was diagnosed with R-ISS II, standard risk IgG kappa myeloma in 2013 after she was found to have amyloid deposits on a gastric mucosal biopsy, which was performed for gastric varix detection. Amyloid typing revealed the presence of AL (kappa)-type amyloid. This patient underwent induction with four cycles of bortezomib, cyclophosphamide, and dexamethasone followed by high-dose melphalan and an ASCT. She achieved complete remission and was started on lenalidomide maintenance until she developed disease progression 29 months after her ASCT. Thereafter, the patient received an additional seven lines of therapy, including IMiD, PI, and anti-CD38 MoAb combinations, and developed penta-refractory disease 9 years after her diagnosis. Due to worsening kidney function, a renal biopsy was performed, which showed kappa light chain amyloid deposits with interstitial fibrosis and tubular atrophy. There was also concern for cardiac amyloid, given persistent atrial fibrillation, a left ventricular longitudinal peak systolic strain of −12%, an abnormal left ventricular geometry with concentric left ventricular hypertrophy, an elevated filling pressure, and elevated NT-Pro BNP (8074 pg/mL) and 5th generation troponin T (22 ng/dL), consistent with Mayo 2012 stage II amyloidosis. The patient subsequently underwent leukapheresis for idecabtagene vicleucel (ide-cel) and received bridging therapy with isatuximab, carfilzomib, and dexamethasone while waiting for ide-cel manufacturing. Once ide-cel CAR T cells were in house, the patient received lymphodepletion chemotherapy with 300 mg/m^2^ of cyclophosphamide and 15 mg/m^2^ of fludarabine (dose reduced by 50% due to an eGFR of 18) given on days −5, −4, and −3. In efforts to mitigate CRS and ICANS, the patient received 10 mg of dexamethasone PO on the day of the ide-cel infusion (day 0), as well as on day +1 and day +2, a CRS mitigation strategy borrowed from cohort 6 of the ZUMA-1 trial [12]. Ide-cel was administered at a dose of 4.46 × 10^6^ cells/kg on day 0. The patient developed treatment-related cytopenias (grade 4 neutropenia and grade 2 anemia), of which neutropenia resolved by day 16, with ongoing residual grade 1 anemia most likely due to renal dysfunction. There was no evidence of CRS, ICANS, infection, cardiac decompensation, or worsening renal dysfunction during hospitalization for CAR T. On day +30 after CAR T, re-staging studies showed evidence of a very good partial response (VGPR); PET-CT showed resolution of any lytic bone disease and the bone marrow was MRD negative (via multi-parametric flow cytometry, 10^−5^). The patient achieved stable renal disease and achieved a cardiac response, as evidenced via a >30% decrease in NT-ProBNP (from 8831 pg/mL to 3167 pg/mL) at 9 months post-CAR T based on validated organ response criteria for AL amyloidosis [13,14]. There was also stable cardiac and renal function. The patient has retained a VGPR for the last 258+ days at the time of this report (Table 1).

## 3. Case 2

A 33-year-old African American male was found to have shortness of breath and persistent tachycardia after admission to the hospital in 2018 for a viral pneumonia. This patient underwent monoclonal protein studies, which revealed a lambda light chain of 52.4 mg/dL (kappa of 1.97 mg/dL with a kappa/lambda ratio of 0.0376) with serum immunofixation showing a monoclonal lambda light chain. Their creatinine level was 1.32 mg/dL. A bone marrow aspirate and biopsy showed 15% lambda-restricted plasma cells, with Congo red stain showing intravascular amyloid deposits. FISH testing revealed evidence of t(11;14). Subsequent amyloid typing showed AL (lambda)-type amyloid. The skeletal survey did not reveal any lytic lesions. An echocardiogram showed a left ventricular longitudinal peak systolic strain of −4%, an ejection fraction of 18%, and biventricular dysfunction, along with bi-atrial enlargement and pericardial effusion. Cardiac MRI showed a markedly diminished global LV function, with an ejection fraction of 20%. Their NT-ProBNP level was 7789 pg/mL, while their high-sensitivity cardiac troponin T level was 80 ng/L, consistent with Mayo 2012 stage IV amyloidosis. He was noted to have NYHA Class II heart failure symptoms and was started on carvedilol, lisinopril, and spironolactone. His ejection fraction subsequently increased to 47%. The patient was treated with bortezomib, cyclophosphamide, and dexamethasone and after three cycles achieved a complete hematologic response, but then developed congestive heart failure exacerbation thought to be related to cardiotoxicity from bortezomib. Over the next 4 years, the patient had disease relapses and received single-agent daratumumab, daratumumab plus lenalidomide, single-agent venetoclax, ixazomib, cyclophosphamide, and dexamethasone. While on ixazomib, cyclophosphamide, and dexamethasone, the patient developed multiple hypermetabolic lytic lesions in their axial skeleton, most concentrated in the bilateral acromion, clavicles, and sternum. Given the progression of their disease manifesting as myeloma lytic bone disease, it was decided to proceed with ciltacabtagene autoleucel (cilta-cel) as the patient had received four prior lines of therapy. The patient subsequently underwent leukapheresis for cilta-cel and received bridging therapy with ixazomib, cyclophosphamide, and dexamethasone plus zoledronic acid while waiting for cilta-cel manufacturing. The patient was closely followed by cardiology for medical management. Once cilta-cel CAR T cells were in house, the patient received lymphodepletion chemotherapy with 300 mg/m^2^ of cyclophosphamide and 30 mg/m^2^ of fludarabine given on days −5, −4, and −3. In efforts to mitigate CRS and ICANS, the patient received 10 mg of dexamethasone PO on the day of the cilta-cel infusion (day 0), as well as on day +1 and day +2, a strategy borrowed from cohort 6 of the ZUMA-1 trial [12]. Cilta-cel was infused at a dose of 0.75 × 10^6^ cells/kg. On day +6, the patient developed grade 3 CRS consisting of fever and hypotension requiring vasopressor support. The patient was admitted to the intensive care unit and started on norepinephrine. The patient received two doses of 8 mg/kg of tocilizumab as well as IV dexamethasone (10 mg) every 12 h for 24 h. The CRS completely resolved within 24 h after receiving CRS treatment, and the patient was downgraded back to the CAR T unit. The patient remained euvolemic throughout hospitalization without heart failure exacerbation. The patient also developed pancytopenia (grade 1 anemia, grade 3 neutropenia, and grade 3 thrombocytopenia), of which thrombocytopenia and neutropenia improved by day +35 and day +69, respectively, with a persistent residual grade 1 anemia. No evidence of ICANS was observed at any timepoint. Day +30 re-staging revealed evidence of disease progression, as PET-CT revealed new lytic hypermetabolic right 8th and left 10th rib lesions. However, there was evidence of MRD negativity (via multi-parametric flow cytometry, 10^−5^) in the bone marrow and no detectable monoclonal proteins in the blood or urine via protein electrophoresis and immunofixation. The patient was started on monthly 120 mg denosumab doses to prevent skeletal-related events. On a day +60 PET-CT, the FDG avidity of these lesions decreased, and a repeat PET-CT at 9 months post-CAR T showed a complete resolution of the FDG avidity of these lytic lesions, and there continued to be no detectable monoclonal proteins in the blood or urine via protein electrophoresis and immunofixation consistent with a deepening of response to a stringent complete response. The patient ultimately achieved a cardiac response, as evidenced by NT-ProBNP decreasing by >30% to 1677 pg/mL at 9 months post-CAR T [14] (Table 1).

## 4. Discussion

These two cases highlight the feasibility of utilizing anti-BCMA CAR T for the treatment of patients with multiple myeloma and concurrent AL amyloidosis, including patients with cardiac and renal involvement. There has been a previous report highlighting the efficacy of the academic, second-generation ARI0002h BCMA CAR T in a patient with concurrent myeloma and AL amyloidosis with renal involvement [15]. To our knowledge, we have presented the first reported cases of commercially available ide-cel and cilta-cel use for the treatment of patients with AL amyloidosis. While both patients had concurrent multiple myeloma, and their myeloma CRAB symptoms were the main indication for treatment with CAR T, their concurrent amyloidosis presented potential challenges to CAR T tolerability due to underlying cardiac and/or renal dysfunction. However, patient selection and optimization of renal and cardiac function prior to CAR T, as well as close monitoring of organ function following CAR T infusion, contributed to the safety of CAR T in these cases. Additionally, our patients were treated with prophylactic corticosteroids in efforts to mitigate CRS and ICANS, and one of our patients underwent modification of lymphodepleting fludarabine doses due to renal dysfunction (Table 2). There are data to support the use of either corticosteroids or tocilizumab for the mitigation of CRS and ICANS with the use of anti-CD19 CAR T for B cell lymphomas; however these data do not exist for multiple myeloma [12,16]. We borrowed the CRS prophylaxis strategy from cohort 6 of the ZUMA-1 trial, where patients with relapsed/refractory large B cell lymphoma received the anti-CD19 CAR T cell axicabtagene ciloleucel (axi-cel) and received once-daily oral 10 mg of dexamethasone on days 0 (before axi-cel), +1, and +2. With prophylactic dexamethasone, no grade 3 or higher CRS occurred, and there was no compromise in clinical efficacy [12].

Early data from a phase I clinical trial (NCT04720313) evaluating the feasibility of the novel, academic anti-BCMA CAR T NXC-201 in patients with relapsed/refractory AL amyloidosis have been reported [17]. Nine evaluable patients (two of which had concurrent multiple myeloma and four of which had Mayo-stage IIIa/IIIb disease), who had received a median of six prior lines of therapy (range: 3–10), achieved a hematologic overall response rate (ORR) of 100% with six CRs, two VGPRs, and one PR. At day 30, all six patients in CR were MRD negative. The median follow-up was 7.3 months (range: 2.5–16.5), and the median duration of response was 5 months (range: 2.5–16.5). Organ responses were observed in six patients. Seven patients experienced CRS that was grade 1 (*n* = 2), grade 2 (*n* = 3), or grade 3 (*n* = 2). The median time to onset of CRS was 2 days (range: 1–3), and the median duration of CRS was 1 day (range: 1–4). There were no instances of ICANS. Within the first 2 weeks, two patients experienced AL-related acute renal failure, and one patient had grade 3 hepatic dysfunction that subsequently resolved. There were no treatment-related deaths [17].

Emerging data in a small number of patients are showing the safety, tolerability, and efficacy of anti-BCMA CAR T in patients with relapsed/refractory AL amyloidosis, including those with advanced stage disease. Importantly, our report and the phase I trial of NXC-201 show that a high proportion of patients treated with anti-BCMA CAR T achieve a MRD-negative state, which is key for patients with AL amyloidosis as MRD negativity has been associated with improved organ responses [18]. The available data, including this report, suggest that anti-BCMA CAR T can be well tolerated by heavily pre-treated AL amyloidosis patients with organ dysfunction, and that CRS is manageable as no treatment-related deaths have been reported. However, how best to optimize organ function prior to and during CAR T therapy and strategies to mitigate CRS remain unknown. More prospective data will be needed regarding the safety, efficacy, and management of CAR T therapy for patients with AL amyloidosis; however, AL amyloidosis is a rare disease, and large controlled clinical trials are difficult to conduct [19]. Perhaps, the field will have to rely on small trials, pooled data, and expert opinions to devise selection criteria for the use of CAR T cell therapy for patients with AL amyloidosis, as improved patient selection and defined eligibility criteria has allowed ASCTs to be used in patients with AL amyloidosis [20,21].

## 5. Conclusions

Anti-BCMA CAR T cell therapy with ide-cel and cilta-cel appears to be a safe and efficacious therapeutic strategy for select patients with concurrent multiple myeloma and AL amyloidosis. Medical optimization of cardiac and renal function prior to CAR T, as well as prevention of CRS, are crucial supportive care interventions to mitigate toxicity. Further evaluations of anti-BCMA CAR T therapy, along with CRS mitigation and patient selection guidelines, are warranted to make CAR T a viable therapeutic option for patients with AL amyloidosis.

## Figures and Tables

**Table 1:** Laboratory and clinical parameters from apheresis until day +30 post-CAR T.

	at T Cell Pheresis	at CAR T Cell Infusion	Day +30
CASE 1			
CRAB symptoms	Present (lytic bone lesions)	Present (lytic bone lesions)	Resolved/none
Kappa FLC (mg/dL)	1.48	2.16	0.1619
Lambda FLC (mg/dL)	1.26	1.25	0.2281
Serum M protein (g/dL)	0.2	ND	ND
Serum immunofixation	IgG kappa	IgG kappa	IgG kappa
Creatinine (mg/dL)	3.41	2.22	3
eGFR (mL/min/BSA)	<15	25	17
Serum LDH (U/L)	303	185	297
B2 microglobulin mcg/mL	15.30	ND	ND
Plasma cells (%) in bone marrow biopsy	ND	ND	MRD negative
NT-proBNP (pg/mL)	8315	8831	ND
Troponin T (ng/L)	27	ND	ND
Echocardiography	LVEF 71%	ND	LVEF 61%
NYHA stage	II	II	I
PET scan	Increased focal metabolic activity seen in the left iliac and sacral bone as well as left ischial tuberosity. Diffuse FDG uptake in the vertebral body, sacrum, and bilateral iliac bones.	ND	No hypermetabolic skeletal foci seen.
Best response (IMWG criteria)	Stable disease	Stable disease	VGPR
CASE 2			
CRAB symptoms	Present (anemia and bone lesions)	Present (anemia and bone lesions)	Present (anemia and bone lesions)
Kappa FLC (mg/dL)	<0.0600	ND	<0.0600
Lambda FLC (mg/dL)	6.13	ND	<0.1372
Serum M protein (g/dL)	ND	ND	ND
Serum immunofixation	ND	ND	ND
24 h Urine monoclonal protein (mg/24 h)	ND	ND	ND
Urine M protein	None	ND	None
Urine immunofixation	Negative	ND	Negative
Creatine (mg/dL)	1.23	1.05	1.35
eGFR (mL/min/BSA)	79	>90	71
Serum LDH (U/L)	271	273	297
Plasma cells (%) in bone marrow biopsy	ND	ND	MRD negative
NT-proBNP (pg/mL)	2095	4685	4122
Troponin T (ng/L)	ND	ND	ND
Echocardiography	LVEF 37%. Global average LV peak systolic strain of −6%.	ND	ND
NYHA stage	II	II	II
PET scan	Multiple hypermetabolic lytic lesions seen in the bilateral acromion, clavicles, and sternum	ND	New lytic hypermetabolic right eighth and left tenthth rib lesions.
Best response (IMWG criteria)	Progressive disease	Stable disease	Progressive disease

FLC: free light chain; ND: not detected or not performed; MRD: minimal residual disease; VGPR: very good partial remission; NYHA: New York Heart Association; LVEF: left ventricular ejection fraction; GFR: glomerular filtration rate; PET: positron emission tomography; IMWG: International Myeloma Working Group; and FDG: fluorodeoxyglucose.

**Table 2:** Highlights of CAR T in two patients with AL amyloidosis.

Drug Modification	CASE 1	CASE 2
Bridging therapy	Isatuximab and carfilzomib-Dexamethasone. Response to bridging: stable disease.	Ixazomib-cyclophosphamide-dexamethasone. Response to bridging: stable disease.
Lymphodepleting chemotherapy	Fludarabine (* 15 mg/m^2^) plus cyclophosphamide (300 mg/m^2^) on days −5, −4, and −3.	Fludarabine ( 30 mg/m^2^) plus cyclophosphamide (300 mg/m^2^) on days −5, −4, and −3.
CRS or neurotox prophylaxis	10 mg of dexamethasone PO added on day 0 (day of CAR T infusion), day +1, and day + 2.	10 mg of dexamethasone PO added on day 0 (day of CAR T infusion), day +1, and day + 2
Management for CRS	Not required.	Two doses of 8 mg/kg of tocilizumab, norepinephrine vasopressor support, and IV dexamethasone (10 mg) q12 h.

* Dose of fludarabine is reduced by 50% due to CKD.

## Data Availability

The datasets generated during and/or analyzed during the current study are not publicly available due to them containing patient personal health information, but they are available from the corresponding author on reasonable request for a de-identified dataset.
